# Examining food insecurity and areas with unmet food needs during COVID-19: A geospatial, community-specific approach

**DOI:** 10.5304/jafscd.2021.103.017

**Published:** 2021-06-24

**Authors:** Kathryn M. Janda, Raven Hood, Amy Price, Samantha Night, William Edwin Marty, Amanda Rohlich, Kacey Hanson, Marianna Espinoza, Alexandra E. van den Berg

**Affiliations:** a. UTHealth School of Public Health; b. Michael and Susan Dell Center for Healthy Living; c. United Way for Greater Austin; d. Central Texas Food Bank; e. City of Austin Office of Sustainability; f. University of Texas at Austin Dell Medical School Population Health Department

**Keywords:** 2-1-1 Calls, Community Health, COVID-19, Pandemic, Food Insecurity, Health Disparities

## Abstract

Food insecurity is a public health issue that has increased in the U.S. since the 2020 COVID-19 pandemic. Understanding how this increase occurs locally is crucial in informing appropriate food insecurity-related responses. Analyzing 2-1-1 call data is one way to examine food insecurity-related needs at a zip code level. The purpose of this work was to: (1) examine overall call trend data to 2-1-1 from March through July 2019 and March through July 2020, (2) examine changes in food need call volume to 2-1-1 during COVID-19 by zip code, and (3) identify areas with unmet food needs during COVID-19 in central Texas. Data for 2-1-1 calls from Travis County zip codes for March through July 2020 were compared to calls for March through July 2019 and categorized by reason for calling. Descriptive statistics and paired t-tests were used to analyze food need calls by zip code and mapped using ArcGIS. Communities with high food call volume and no emergency food assets located within the zip code were categorized as areas with unmet food needs. Results indicated there were more overall calls to 2-1-1 in 2020 (*N*=37,572) than in 2019 (*N*=28,623), and significantly more food need calls in 2020 than in 2019 (*p*<0.01). Eastern Travis County, a racially and ethnically diverse and lower-income area, had the largest increase in food need calls. Two zip codes were identified as having unmet food needs, which informed the strategic placement of emergency food assets. This study illustrates how 2-1-1 data can result in rapid translation of research to policy and program implementation.

## Introduction and Literature Review

Food insecurity is defined as the inconsistent access to a sufficient amount of food to live an active and healthy life (Pinstrup-Andersen, 2009). While considered a wealthy nation, the United States had high rates of household food insecurity prior to the outbreak of the novel coronavirus (COVID-19), with 11.1% of households identifying as food insecure in 2018 (United States Department of Agriculture Economic Research Service [USDA ERS], 2019). Since the start of the COVID-19 pandemic, the prevalence of food insecurity has increased dramatically in the U.S. (Niles et al., 2020; Schanzenbach & Pitts, 2020; Wolfson & Leung, 2020). Research conducted by Northwestern University found that domestic food insecurity has more than doubled to 25.5% during the COVID-19 pandemic (Schanzenbach & Tomeh, 2020). Similar to national statistics, food insecurity prevalence in Texas has doubled, with over 28% of Texans identifying as food insecure in April through June 2020 (Schanzenbach & Tomeh, 2020). To address the rising rates of food insecurity, local officials need rapid and local data to inform local policies and strategic implementation of food insecurity mitigation programs. The purpose of this paper is to describe how a novel data collection method can be used to rapidly identify areas experiencing unmet food needs and inform programming and policies during the pandemic.

### Food Insecurity and Disparities Prior to and During COVID-19

Food insecurity is often the byproduct of poverty or economic disadvantage and does not occur in isolation (Bhattacharya et al., 2004; Finney Rutten et al., 2010; Gundersen et al., 2011). Additionally, food insecurity prevalence has historically increased during high unemployment and/or economic recession (Andrews & Nord, 2009; Loopstra et al., 2016). Some scholars identified that the combination of high unemployment rates, economic down-turn, stay-at-home orders, school closures (and consequently the reduced offering of school nutrition programs), closure and/or limited hours of food retail, and social distancing policies during COVID-19 have had a particularly dramatic impact on food insecurity (Choudhury et al., 2020; Dunn et al., 2020; Laborde et al., 2020; Pérez-Escamilla et al., 2020; Shanks et al., 2020; Wolfson & Leung, 2020).

Before the pandemic, people of color and low-income households were more likely to be food insecure than people who lived in white and/or more wealthy communities (Hernandez et al., 2017; Odoms-Young & Marino, 2018; Seligman & Schillinger, 2010). Additionally, communities of color and low-income areas are more likely to have limited geographic food access, meaning that they typically do not have healthy food retail options in their neighborhoods and have to travel farther to access food (Alkon & Agyeman, 2011; Larson et al., 2009; Morales, 2011; Walker et al., 2010). These disparities are evident in central Texas, where eastern Travis County, largely due to historic redlining practices, has generally had a larger population of Black and Hispanic communities, lower median household income, fewer healthy food retail opportunities, and a higher prevalence of food insecurity than western Travis County (City of Austin Office of Sustainability, 2019; City of Austin Travis County Health and Human Services Department, 2015; Huggins, 2017; United Way of Central Texas, 2019; U.S. Census Bureau, 2018).

Since the start of the COVID-19 pandemic, national and state-level food insecurity data suggest that disparities between racial and ethnic groups are increasing (Morales et al., 2020). Individuals who identify as Black (34.9%) and Hispanic American (34.0%) have been found to have a much higher prevalence of food insecurity during COVID-19 than white Americans (25.5%) at the national level (Schanzenbach & Tomeh, 2020). Furthermore, state-level analyses using Census Pulse Survey data found that Black (35.2%) and Hispanic (33.2%) Texans have a higher prevalence of food insecurity than White Texans (21.6%) (Schanzenbach & Tomeh, 2020). These findings demonstrate that pre-existing food insecurity disparities could be widening during the pandemic; however, there is limited data about food insecurity rates at a local or zip code level. The smallest geographic unit of analysis for food insecurity data during the pandemic has been at the county-level, and it is projected that food insecurity in Travis County has risen significantly during the COVID-19 pandemic (Gundersen et al., 2020). However, determining food insecurity prevalence at a more local level, such as by zip code or census tract, is often only available in national data sets and takes years to become available (Coleman-Jensen et al., 2018). Thus, community-specific food insecurity data is necessary to ensure that all high need areas have additional food assets available during the pandemic and that pre-existing disparities do not widen.

One solution for decreasing food insecurity is to connect individuals who have emergency and chronic food needs to resources that address food insecurity, such as food banks, food pantries, soup kitchens, the Supplemental Nutrition Assistance Program (SNAP, formerly known as food stamps), and hotlines or call lines that can connect individuals to needed resources (Bacon & Baker, 2017; Boyum et al., 2016; Linnan, 2012; O’Connell et al., 2008; Robaina & Martin, 2013).

### Food Insecurity Resources and the United Way 2-1-1 Call Line

One resource that has successfully facilitated network building and resource referrals for individuals in need for the last two decades has been the United Way’s 2-1-1 call line program (Daily, 2012). Established in 2000 by the Federal Communications Commission (FCC), the network of 2-1-1 call line programs became operational in all 50 states and Puerto Rico during the next decade (Daily, 2012; Linnan, 2012). By 2018, 2-1-1 call lines were considered a well-established resource for the community throughout the country. For example, in 2018, the United Way for Greater Austin received over 50,000 2-1-1 calls, of which approximately 11% regarded food insecurity-related issues (Janda et al., 2020). United Way considers all callers to 2-1-1 to be clients of United Way.

The United Way for Greater Austin operates the 2-1-1 call line in Travis County. Since the start of the pandemic, United Way has helped disseminate information pertaining to COVID-19 through the central Texas region (United Way for Greater Austin, 2020a, 2020b). In Texas, 2-1-1 is considered a key COVID-19 information resource publicized by billboards, radio, and many government officials’ public addresses, including Governor Abbott and City of Austin officials (Weber, 2020). Additionally, United Way for Greater Austin kept consistently updated records on the location and availability of emergency food assets and open resources during the COVID-19 pandemic (United Way for Greater Austin, 2020b).

United Way for Greater Austin/2-1-1 also collaborated with Austin Public Health and the City of Austin Office of Sustainability’s Food Policy Team (which existed prior to COVID-19) to help coordinate responses to local food systems issues. The Food Access Coordination Taskforce, led by the City of Austin Office of Sustainability, became crucial in this coordination and consists of approximately two hundred individual contacts representing city and county departments, school districts, nonprofits, and community-based organizations. The taskforce met weekly to share updates on organizational operations, identify areas of need (including opportunities for collaboration and directing resources), and develop a longer-term strategy to address the anticipated increase in food access needs throughout the community after the pandemic. However, the taskforce realized that it needed more data to understand how food insecurity was changing at a community level throughout the county and to inform policy and program implementation.

### Gaps in the Literature and Community Needs

At the start of the pandemic, there were no datasets or reports available that included data regarding what areas of Travis County were experiencing especially high food insecurity rates. 2-1-1 call data were identified by the City of Austin and other collaborators as a potentially valuable source for information regarding food needs that could provide zip-code level data and could be paired with geographic food asset location data. Additionally, these data could fill a notable gap to better inform policies and programs to strategically place assets in areas with high food insecurity needs. Thus, in early April 2020, the City of Austin Office of Sustainability contacted UTHealth School of Public Health in Austin and Dell Medical School to utilize 2-1-1 call data to examine changes in food needs in Travis County and identify areas with unmet food needs in Austin during COVID-19.

### Research Objective

The objective of this work was to build a transdisciplinary collaboration that could: (1) examine overall call trend data to 2-1-1 in March–July 2019 and March–July 2020, (2) examine changes in food need call volume to 2-1-1 during COVID-19 by zip code, and (3) identify areas with unmet food needs during COVID-19 in Travis County, Texas.

## Applied Research Methods

### Study Design and Study Area

The study design was a natural experiment and utilized 2-1-1 call data from Travis County, Texas, during March–July 2019 and March–July 2020, with 2019 dates considered a pre–COVID-19 comparison. Participants were callers to the 2-1-1 call line from March 1 through July 31, 2019, and March 1 through July 31, 2020. The 2020 time frame was selected because awareness of COVID-19 gained traction in early March 2020; federal, state, county, and city COVID-19 policies were announced, and the closure of universities, schools, and large events all started occurring in Travis County (Weber, 2020).

The sample was restricted to those who made calls to the United Way for Greater Austin in the aforementioned time frame from Travis County, Texas. Callers who did not specify their county of residence, who did not specify their zip code of residence, or who reported a post office box–only zip code as an address were dropped from the analysis. Data about emergency food assets were obtained from collaborators at the United Way for Greater Austin and the City of Austin Office of Sustainability. This study was approved and considered exempt by the Institutional Review Board (IRB) at UTHealth School of Public Health (HSC-SPH-20-0518) because the callers were unidentifiable, and there was no way to follow-up with callers. Furthermore, the IRB determined that consent was implied because all callers to the 2-1-1 call line are informed that information regarding the nature of their call will be included in 2-1-1’s call log and records.

### Examination of Overall and Food Need 2-1-1 Call Data Methodology

#### Call data categorizations

All 2-1-1 calls used in this analysis were categorized into thematic groups based upon the resources requested by the caller. The thematic categories were finance and unemployment, food needs, health and mental health, housing, transportation, utilities, and other related calls. For this analysis, food need calls to the 2-1-1 call line served as a proxy for food. Examples of food need calls included callers looking for food pantries, soup kitchens, food banks, food assistance (such as the Supplemental Food Nutrition Assistance Program and Pandemic-EBT), and other food-specific resources. Demographic data, including the caller’s zip code, sex, and language spoken during the call, were noted in the call log and were self-reported by the caller. No other identifiable data were included in the call log.

#### Call data analysis

Descriptive statistics and paired t-tests were utilized for this analysis. Frequencies were calculated for March–July 2019 and March–July 2020 by call categories, demographic information included in the 2-1-1 call log, and zip code. To contextualize call trends longitudinally during the pandemic, overall and food need call volumes were also analyzed by week. Paired t-tests by zip code were calculated to determine if there were statistically significant differences in the mean number of food need calls by zip code in March–July 2019 to March–July 2020. The change in the percent of food need calls by zip code was calculated and then mapped using ArcGIS (ESRI, 2019). All frequencies and t-tests were run utilizing Stata (version 14) (StataCorp, 2015), graphs were made using R (R Team, 2017), and maps were created with ArcGIS (ESRI, 2019; StataCorp, 2015; R Team, 2017).

### Methodology for Identifying Areas with Unmet Food Needs

Location of emergency food assets was needed to identify areas with unmet food needs. United Way for Greater Austin and the City of Austin Office of Sustainability provided information on the location and addresses of these assets. These locations were consistently updated during March–July 2020 to reflect potential changes in operation. These locations were then geocoded using ArcGIS and included in the analysis for identifying zip codes with unmet food needs (ESRI, 2019).

More specifically, zip codes with unmet food needs were determined by the convergence of high food need call volume, a high proportion of food need calls, a large change in food call volume in the zip code from 2019 to 2020, and a lack of emergency food assets present in the zip code. Zip codes with above-average food need call volumes (with the average determined to be 191 calls during March–July 2020) were classified as having a high food need call volume. Zip codes with an above-average percentage of food need calls for Travis County (i.e., more than 29%) during March–July 2020 were classified as having a high proportion of food need calls. A large change in food call volume within a zip code from 2019 to 2020 was determined as being a greater than 10% increase.

## Results

### 2-1-1 Call Trends during COVID-19

The total sample consisted of 28,623 calls during March–July 2019 and 37,572 calls during March– July 2020 (Table 1). From March through July 2019, health and mental health was the most common reason for calling 2-1-1 (30.07%), followed by food need calls (23.29%). During March–July 2020, food needs were the most common reason for calling 2-1-1 (35.13%), followed by health and mental health calls (27.49%). During both periods, callers were predominantly female (2019: 72.45%, 2020: 70.14%) and spoke English (2019: 86.48%, 2020: 82.71%) or Spanish (2019: 13.29%, 2020: 17.13%). The 2-1-1 call line navigators do not ask about the caller’s race and/or ethnicity and income level; therefore, that information could not be provided. However, information regarding sociodemographic composition (including race/ethnicity, income level, etc.) by zip code for Travis County is provided by the 2018 American Community Survey and can be found in Figure A in the Appendix.

**Figure 1.**
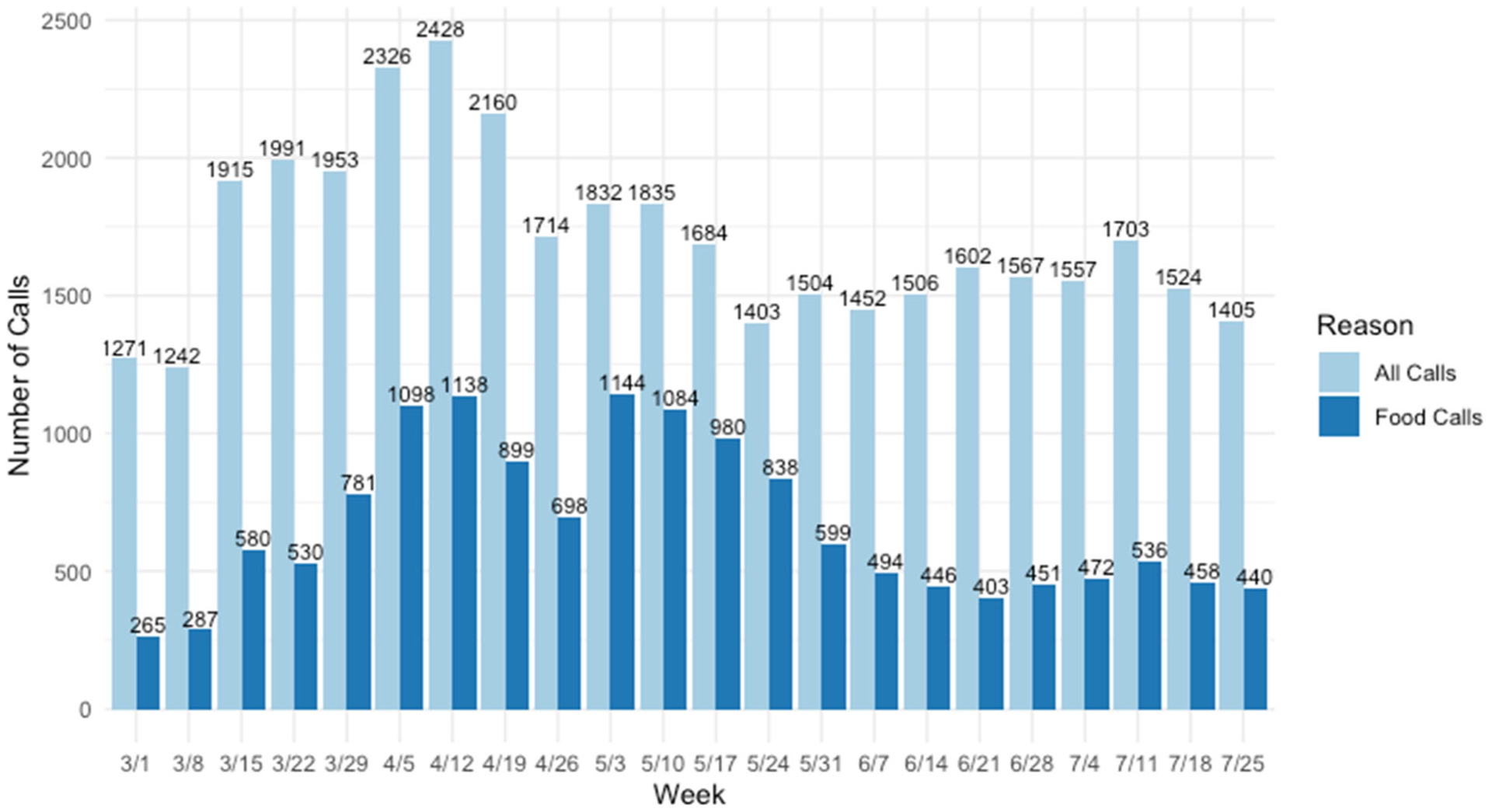
Overall and Food Call Data by Week, March 1–July 31, 2020

To better contextualize the call trends longitudinally during the COVID-19 pandemic, overall and food need call data were also analyzed by week (Figure 1). Overall call volume increased sharply during the middle of March, peaked the week of April 12, 2020, and then fluctuated with smaller increases in overall call volume at the end of April and beginning of May, and in early July. Food need call volume also rose sharply in mid-March, peaked the week of May 3, 2020, and consistently declined with small increases in early July. The large increase in food need calls in April and May was mostly due to calls regarding food assistance benefits, such as SNAP.

### Changes in Food Calls to 2-1-1 during COVID-19 by Zip Code

During March–July 2019 and March–July 2020, callers to 2-1-1 resided in 69 zip codes of Travis County. Results from the paired t-tests found that there were statistically significant differences in food need call volume (t=−4.93, df=68, *p*<0.01) and percentage of food need calls (t=−5.77, df = 68, *p*<0.01) in zip codes from 2019 to 2020. Thus, there were significantly more food need calls to 2-1-1 across Travis County during the COVID-19 pandemic than there were during the same months in 2019.

**Figure 2.**
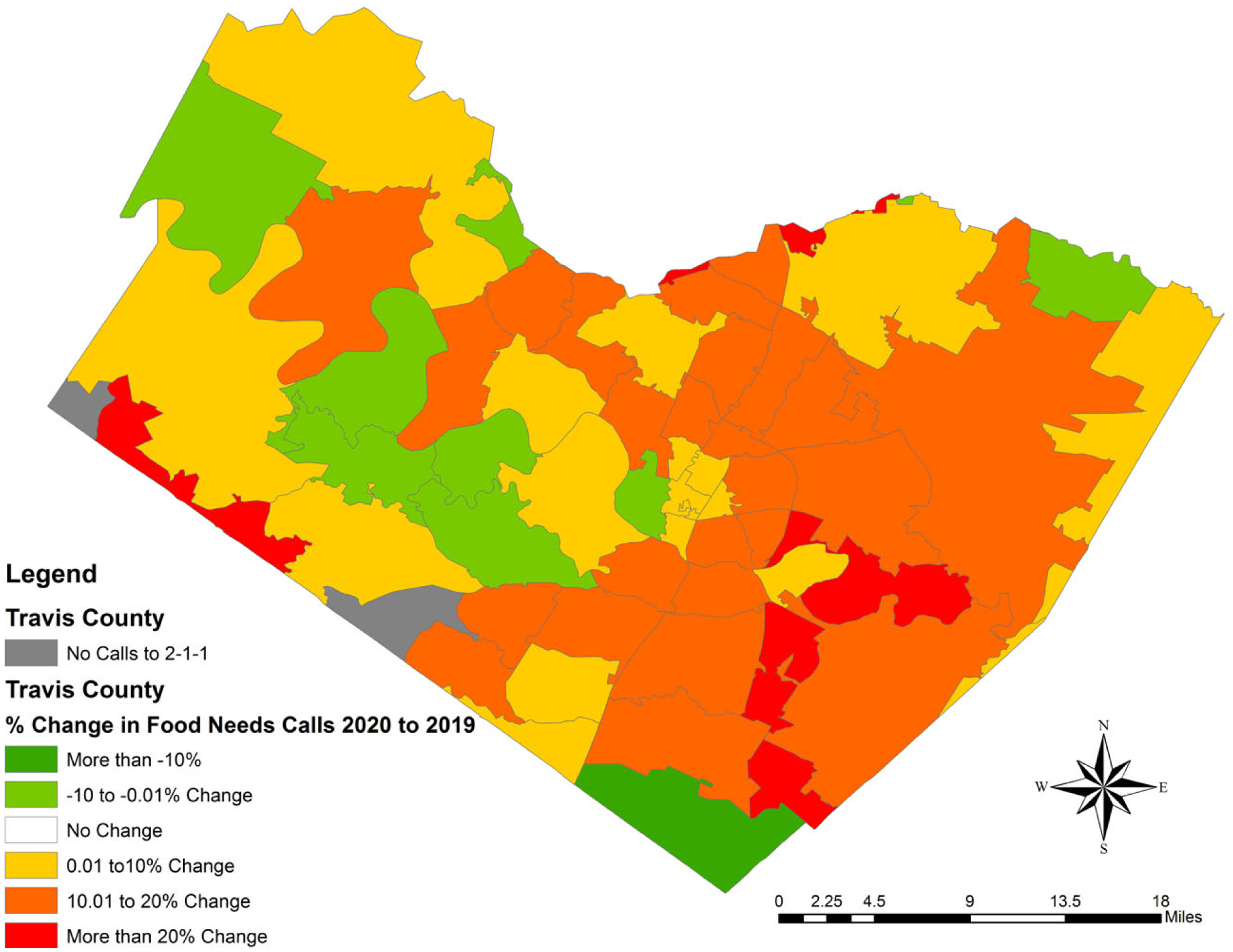
Changes in Percent of Food Need Call Volume by Zip Code, March–July 2019 to March–July 2020

The changes in percent of food need calls by zip code were calculated and then mapped (Figure 2). Over 78% of zip codes (*n*=54) saw an increase in the percentage of food need calls made to 2-1-1 during 2020 compared to 2019. Additionally, over half the zip codes (*n*=36) saw an increase greater than 10% in the percentage of food need calls made in the zip code during March–July 2020 compared to March–July 2019, as shown in red and scarlet in Figure 2. Eastern Travis County has more zip codes in dark orange and red than western Travis County.

### Identification of Areas with Unmet Food Needs

The final component of this analysis was to identify areas with unmet food needs during the pandemic in Travis County. Geographic analyses of food need calls were compared to locations of open emergency food assets monthly. Zip codes with potential unmet food needs were identified if they had a high overall food need call volume (more than 191 food need calls), a high proportion of food need calls (over 29% of calls regarded food needs in the zip code), an over 10% increase in volume and percent of food need calls in March–July 2020 compared to March–July 2019, and lack of an operating emergency food asset located in the zip code from March–July 2020. Two zip codes met the aforementioned criteria. These zip codes were identified by City of Austin officials and other stakeholders as needing a strategically placed food asset during the pandemic.

## Discussion

### Summary and Implications of Findings

#### 2-1-1 overall and food need call level findings and implications

The purpose of this component of the analysis was to examine overall call trend data to 2-1-1 during March–July 2019 and March–July 2020 and to examine changes in food need call volume to 2-1-1 during COVID-19 by zip code. The results showed higher overall call volume and significantly higher food need call volume throughout Travis County during the COVID-19 pandemic. While there was an increase in overall call volume, the category with the greatest percent increase from 2019 to 2020 was food need calls. Additionally, results from the weekly call analysis shed greater insight on public response to key policy announcements and reports about the prevalence of COVID-19 cases and hospitalizations. The first “Stay Home, Stay Safe” order for Austin and Travis County was announced in late March and was extended in mid-April. Interestingly, the rise in call volume in June and July matches the reporting of a spike in cases in the Travis County area during that time (City of Austin, 2020; Osborn, 2020; Weber, 2020). Additionally, the peak of food need calls in early May coincides with the first announcement about Pandemic-EBT (Office of the Texas Governor, 2020). Thus, the results demonstrate an increase in calls to 2-1-1 during COVID-19 compared to before the pandemic and that local policy changes and announcements correlate to a rise in 2-1-1 call volume.

While an increase in food need calls using 2-1-1 data is not a precise or validated measurement of the prevalence of food insecurity, the results demonstrate that food insecurity–related issues and needs are being experienced by a growing number of United Way clients. Additionally, this increase is consistent with the projected increases in food insecurity calculated by Feeding America and the increased prevalence of food insecurity experienced by Americans and Texans found in the Census Pulse Survey analysis (Feeding America, 2020; Schanzenbach & Tomeh, 2020). This dramatic rise in food insecurity is alarming, given that it has taken 10 years to recover to pre–Great Recession levels (Gundersen et al., 2020).

Results show a greater increase in food need call volume and the change in the proportion of food need calls from 2019 to 2020 in eastern Travis County than in western Travis County. This is an important distinction because eastern Travis County has been identified as a historically under-served and racially and ethnically diverse area with a higher prevalence of food insecurity than western Travis County (U.S. Census Bureau, 2018). Thus, these findings demonstrate that COVID-19 could be widening the food-insecurity disparities in Travis County and the greater Austin area that existed before the pandemic.

#### Implications of identification of areas with unmet food needs

The third aim of this analysis was to identify areas with unmet food needs during the COVID-19 pandemic in Travis County. This analysis was also able to provide timely and zip-code level recommendations to government officials and nonprofit organizations to implement programs and strategies to potentially ameliorate food insecurity in areas with unmet food needs. The researchers (KJ, AB) disseminated their findings through monthly reports to the City of Austin (SN, WEM, AR), United Way for Greater Austin (AP), Dell Medical School (KH, ME), the City of Austin Food Access Coordination Taskforce, and other collaborators. They also recommended strategically placing an emergency food asset in the two zip codes with unmet food needs. Due to sharing these results and findings, a local nonprofit organization has strategically placed an emergency food asset in one of the identified areas as of late September 2020, and other county and city officials have utilized these data as rationale for additional funding for existing but overburdened resources. Also, this work has informed where other studies should concentrate sampling for their research (KH, ME). Future research should dive deeper into what factors contribute to these communities’ increased proportion of food need calls and how this informs additional policies.

#### Implications of methodological approach

This methodology provided a rapid translation of research to policy and program implementation and has implications for food insecurity research. As discussed previously, food-insecurity data often takes years to obtain, analyze, and disseminate, especially at a granular level such as by zip code. This methodology has enabled close to real-time analysis of food needs at a countywide and zip-code level, resulting in the ability to strategically place additional emergency food assets in areas with unmet food needs. This methodology has potential for implementation across the U.S., given the nationwide presence of United Way and 2-1-1 call lines to provide local insight into how food insecurity is changing during COVID-19.

### Limitations of Study

There are certain limitations to this study. There are potential threats to the validity of this sample, specifically selection bias and limited generalizability. Since inclusion in this sample required an individual to be aware of and call the 2-1-1 line operated by United Way for Greater Austin, this sample is most likely predominantly low-income, resulting in selection bias. While there were campaigns to promote the utilization of 2-1-1 during this period, it is still unlikely that all Travis County residents experiencing food insecurity were calling 2-1-1 during this time. The sample was predominantly female and English-speaking; therefore, these findings may not be generalizable to all Travis County residents. This was a natural experiment, and so a randomly selected sample was not possible to obtain. While the findings are generalizable to the population of 2-1-1 callers in Travis County, future research could expand on these findings with a more representative sample from the county.

Additionally, food need calls were used as a proxy for food insecurity–related issues, which, while used in previous analyses with 2-1-1 call data, is not a validated food-insecurity measurement (Janda et al., 2020). Adding validated food insecurity items as part of the 2-1-1 call protocol, such as the USDA 18-item measure, or two-item food insecurity screener, would result in a more precise measurement of the prevalence of food insecurity among callers (Gundersen et al., 2017; Jones et al., 2013; Pinstrup-Andersen, 2009). Future work should explore rising trends in food insecurity and widening disparities during COVID-19 with a more representative sample to minimize selection bias and should utilize validated food-insecurity survey instruments to more precisely measure food insecurity.

## Conclusions

Research indicates that food insecurity is increasing during COVID-19 throughout the U.S. Results from this study indicate that these projected increases occur at the local level in central Texas and potentially widen pre-existing disparities during the pandemic. Additionally, this study demonstrates that cross-sector collaboration and utilization of this methodology of analyzing 2-1-1 call data at the zip-code level can result in rapid translation of research into policy. Thus, greater research and transdisciplinary and sector partnership are needed to gain a more nuanced understanding of how food insecurity is increasing at a local level and to subsequently inform effective program and policy implementation.

## Figures and Tables

**Table 1.** Call-Level Descriptive Statistics for March–July 2019 and March–July 2020

	March-July 2019		March-July 2020	
Overall Call Volume	*N*=28,623	Percent	*N*=37,572	Percent
**Call Need Categories**				
Food	6,667	23.29%	13,197	35.13%
Health/Mental Health	8,606	30.07%	10,327	27.49%
Housing	5,086	17.77%	7,385	19.66%
Finance/Unemployment	2,810	9.82%	3,942	9.02%
Utilities	2,129	7.44%	2,409	6.41%
Transportation	1,689	5.90%	512	1.36%
Other	6,073	21.22%	6,688	17.8%
***Demographics***				
**Gender**				
Male	7,539	26.34%	10,644	28.33%
Female	20,737	72.45%	26,353	70.14%
Uncertain	347	1.21%	575	1.53%
**Preferred Language**				
English	24,753	86.48%	31,075	82.71%
Spanish	3,804	13.29%	6,436	17.13%
Other	66	0.23%	61	0.16%

**Figure A.**
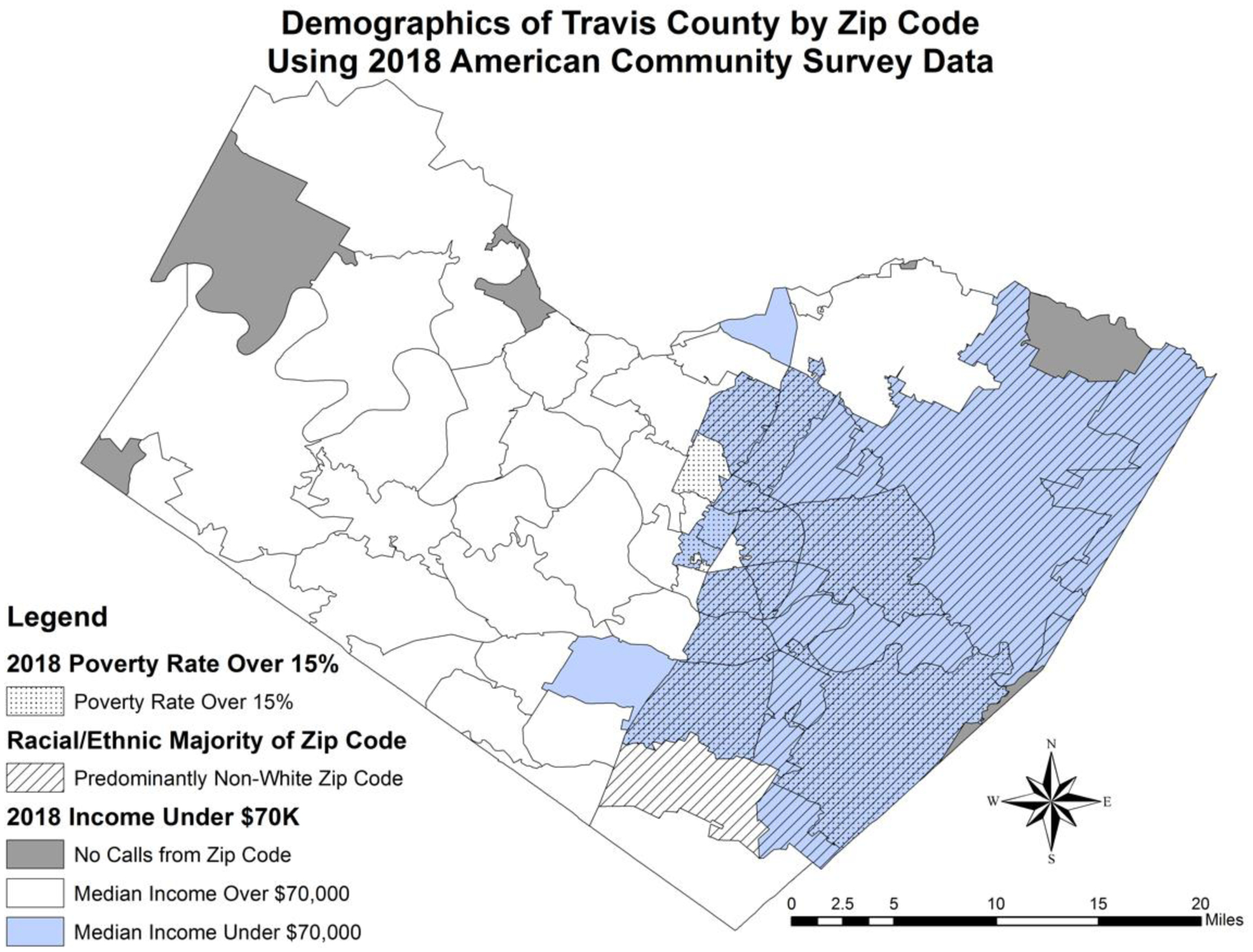
Map of Demographic Data of Travis County by Zip Code Using 2018 American Community Survey Data
